# Recurrent episodic vertigo secondary to hyponatremic encephalopathy from water intoxication

**Published:** 2014-10

**Authors:** Jiann-Jy Chen, Hsin-Feng Chang, Dem-Lion Chen

**Affiliations:** *From the Department of Neurology (Chen J), Neuro-Medical Scientific Center, Buddhist Tzu Chi General Hospital, Taichung Branch, the Faculty of Chinese Medicine (Chang), College of Chinese Medicine, China Medical University, Taichung, and the G-Home Clinic (Chen D), Kaohsiung, Taiwan*

Oral water intoxication is occasionally noted in infants, the elderly, psychotics, or even healthy adults. Patients with impaired free water excretion due to a systemic disease, or medications are also at risk for water intoxication. In some cases, hyponatremic encephalopathy can lead to neurologic morbidity and mortality. The signs and symptoms are primarily neurologic, including headache, anorexia, nausea, cramping, vomiting, personality changes, stupor, seizures, coma, respiratory arrest, and even death.^1^ The severity depends on the rapidity of onset and the extent of hyponatremia (serum sodium levels less than 130 mEq/L).^2^ However, frequent water intoxication related episodic vertigo is scarcely reported in the literature, and here we present such a case, and the subsequent treatment.

Over a 4-year period (**Table 1**) an 80-year-old female presented to the emergency department 14 times with vertigo and hypo-osmolar hyponatremia due to water intoxication. The water intoxication developed after she developed xerostomia with associated polydipsia. She weighed 46.5 kg with a height of 152 cm, and a body mass index of 20.1 kg/m^2^. She had a 10 year history of type II diabetes mellitus, and hypertension for 10 years. Her medications included acarbose (Glucobay 50mg, Bayer Schering Pharma AG, Leverkusen, Germany), glimepiride (Amaryl 2mg, Handok Pharmaceuticals Co., Chungcheongbuk-do, Korea) and valsartan (Diovan 160mg, Novartis Farmaceutica S.A. Stein, Switzerland). She denied alcohol, cigarettes, coffee, or areca chewing. The vertigo with oscillopsia, nausea, and vomiting would develop after she consumed a pitcher of water (4 liters) in several hours. Changing position or head rotation increased the symptoms, and lying in supine position decreased the symptoms. Her vital signs, physical exam, mental status, and neurologic exam were always normal with the exception of a spontaneous leftward horizontal beating nystagmus. Electrocardiograms and blood chemistries were unremarkable with the exception of hyponatremia (**Table 1**). The vertigo always resolved following the correction of hyponatremia.

**Table 1:** Sodium and osmolarity of each symptom attack in a patient with water related episodic vertigo.

Symptom attack (timeline)	Sodium (mmol/L)	Osmolarity (mOSM/kg)
1^st^	125	260.2
2^nd^ (4 months)	121	251.4
3^rd^ (17 months)	126	262.2
4^th^ (18 months)	118	246.9
5^th^ (19 months)	125	263.5
6^th^ (29 months)	125	258.9
7^th^ (30 months)	129	269.1
8^th^ (34 months)	129	267.3
9^th^ (35 months)	129	268.2
10^th^ (39 months)	122	257.5
11^th^ (40 months)	123	256.3
12^th^ (40.5 months)	118	248.5
13^th^ (43 months)	124	257.7
14^th^ (57 months)	120	249.1
Normal reference	135–144	285.0–295.0

At presentation to the emergency department with her fourteenth symptomatic attack of water intoxication and vertigo, she did not have any somatosensory symptom. Judgment, orientation, memory, attention, and calculation were all intact. There was no headache, dysphonia, aphasia, dysarthria, dysphagia, blurred vision, diplopia, hypesthesia/thermanesthesia, dysmetria, hemiparesis, abasia, or astasia. Extraocular movements were intact. On straight ahead gaze, a spontaneous leftward horizontal beating nystagmus without a torsional component in the counter clockwise direction was observed, which was not suppressed by fixation and increased in leftward gaze in amplitude and frequency. Such a pure horizontal nystagmus is usually not found in a peripheral vestibular failure, and might indicate a central origin. After horizontal head shaking, the nystagmus was more pronounced. The clinical head-impulse test of the horizontal semicircular canals was pathological to the right but not the left. Testing horizontal and vertical saccades and smooth pursuit eye movements revealed no further deficits. The Romberg test showed a rightward deviation, the Fukuda stepping test could not be performed due to communication problems. She was able to ambulate with help, but had increased unsteadiness while ambulating with the eye closed. As in tandem gait, she had mild lateropulsion to the right side. There was no somatosensory or motor deficit. She did not have orthostatic hypotension. She did not submit to electronystagmogram or caloric test.

**Figure 1:**
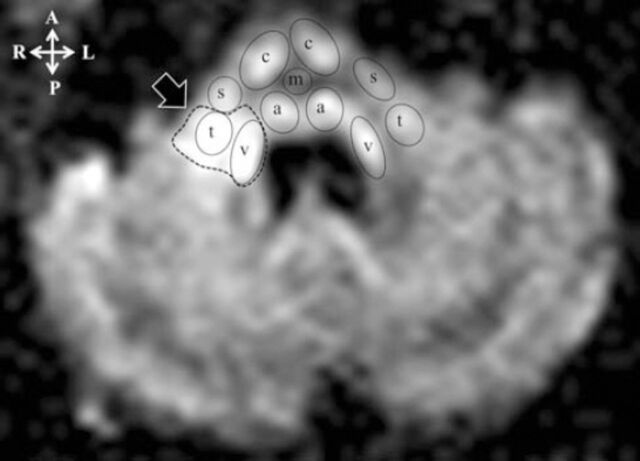
Diffusion weighted MRI (repetition time/echo time/excitation: 9,212/144/1) demonstrated a hyperintense area in the right-sided upper medulla (filled arrow). The hyperintense area (dotted line circle) involved the right vestibular nucleus (v) and spinal tract of the trigeminal nerve (t). c - corticospinal tract, m - medial lemniscus, a - abducens nucleus, s - anterior spinocerebellar tract.^6^

An MRI demonstrated brain atrophy instead of any old infarction on T1 and T2 images with fluid-attenuated inversion recovery (FLAIR) and a hyperintense area in the right-sided upper medulla on the diffusion weighted image (DWI) (**Figure 1**). Time-of-flight magnetic resonance angiogram was unremarkable. Her serum sodium was 120 mmol/L with an osmolality of 249.1 mOSM/kg. Following treatment with a 100 mL 3% saline bolus intravenously over one hour, the vertigo and nystagmus subsided with serum sodium rechecked of 132 mmol/L, and an osmolality was 273.6 mOSM/kg. Following discharge, she was instructed to keep a water diary and to add a teaspoon of salt daily to a 600 ml bottle of sport drink. Over the following 2 years she was without further episodes of vertigo or water intoxication, but she eventually died of pneumonia.

The presenting symptoms of hyponatremic encephalopathy are variable, including headache, nausea, vomiting, and lethargy. These symptoms can easily be overlooked as they occur in a variety of conditions. Also, the disease progression can be abrupt. Possible mechanisms for the symptoms of hyponatremic encephalopathy due to cytotoxic cerebral edema include: 1) diffuse brain parenchyma swelling compresses the supplying vessels and reduces cerebral blood flow, contributing to ischemia;^3^ 2) an acute drop in serum sodium causes osmotic disequilibrium and a shift of water into brain cells despite the intact blood-brain barrier; 3) others include the energy failure of Na+/K+ ATPase pump, excessive increase of membrane ion permeability or excessive release of excitatory amino acids.^4^,^5^ A brain CT is not sensitive enough to detect mild cerebral cytotoxic edema of hyponatremic encephalopathy, which can be detected by DWI MRI.^4^,^5^ Her T1 weighted and T2 FLAIR MRI demonstrated brain atrophy rather than any old infarction, but DWI demonstrated a hyperintense area in the right-sided upper medulla (**Figure 1**).

In the present case, the patient suffered from 14 episodes of symptomatic hyponatremia over a 4 year period due to water intoxication to quell the thirst of xerostomia. She had no evidence of volume depletion or fluid overload and this appeared to be acute euvolemic hyponatremia (onset less than 48 hours) with an increase in total body water and no change in body sodium storage,^1^ and hence contributed to the hyponatremic encephalopathy of the right-sided upper medulla. As there was no old lacune left in her brain, the prior 13 vertigo episodes were not attributable to ischemia stroke; hence, cytotoxic brain edema was preferable to ischemia stroke in the fourteenth vertigo episode. According to her DWI MRI, the cytotoxic brain edema involved the right vestibular nucleus and spinal tract of the trigeminal nerve in the right-sided upper medulla (**Figure 1**). Although she did not have any ipsilateral trigeminal sensory defect, the impairment of the right vestibular nucleus contributed to 1) right vestibulopathy manifested as a pathologic head-impulse test during rightward head rotation, and 2) predominance of left vestibular function manifested as leftward horizontal beating gaze nystagmus, and rightward tilting Romberg test and tandem gait tests; furthermore, the leftward horizontal beating nystagmus could be aggravated by head shaking test.

The therapy for hyponatremic encephalopathy is the intravenous administration of 3% sodium chloride (513 mEq/L). Correction over 25 mEq/L in the first 24-48 hours would lead to cerebral osmotic demyelination, a safe correction of hyponatremia was suggested at 6-15 mEq/L in 24 hours or 20 mEq/L in 48 hours depending on the setting of hyponatremia; however, patients with acute symptomatic hyponatremia have a minimal risk of developing cerebral osmotic demyelization, and insufficient therapy rather than overcorrection has resulted in the majority of the associated morbidity.^5^ In the present case, the hyponatremia was corrected in a controlled setting in the emergency department. Following her final episode she was successfully managed by monitoring her fluid intake in conjunction with salt supplementation. She has been without further episodes of vertigo or water intoxication in the following 2 years before she died of pneumonia.

Recurrent episodic vertigo might result from cervical origins, peripheral vertigo, vestibular migraine, vertebrobasilar artery insufficiency, intracranial hypotension, intracranial hypertension, or even drug side effects. Our patient was on regular acarbose, glimepiride, and valsartan for 10 years so side effects of these drugs were unlikely to be the cause of the symptoms of vertigo that began in the past 4 years.

In conclusion, water intoxication with hyponatremic encephalopathy, although unusual, should be a differential diagnosis of recurrent episodic vertigo. The diagnosis depends on the high index of suspicion and immediate treatment is recommended to prevent further morbidity or mortality.
